# Goethite–titania composite: disinfection mechanism under UV and visible light†

**DOI:** 10.1039/c8ra08412b

**Published:** 2019-01-21

**Authors:** Rosalina Lara-Rico, Elia M. Múzquiz-Ramos, Claudia M. López-Badillo, Ulises M. García-Pérez, Brenda R. Cruz-Ortiz

**Affiliations:** Universidad Autónoma de Coahuila, Facultad de Ciencias Químicas Blvd. V. Carranza s/n Col. República Ote. CP 25280 Saltillo Coahuila Mexico b.cruz@uadec.edu.mx; Universidad Autónoma de Nuevo León, Facultad de Ingeniería Mecánica y Eléctrica, Centro de Investigación e Innovación en Ingeniería Aeronáutica Carretera a Salinas Victoria Km 2.3 Apodaca Mexico

## Abstract

Goethite–titania (α-FeOOH–TiO_2_) composites were prepared by co-precipitation and mechanical milling. The structural, morphological and optical properties of as-synthesized composites were characterized by X-ray powder diffraction, scanning electron microscopy and UV-Vis diffuse reflectance spectroscopy, respectively. α-FeOOH–TiO_2_ composites and TiO_2_-P25, as reference, were evaluated as photocatalysts for the disinfection of *Escherichia coli* under UV or visible light in a stirred tank reactor. α-FeOOH–TiO_2_ exhibited better photocatalytic activity in the visible region than TiO_2_-P25. The mechanical activation increased the absorption in the visible range of TiO_2_-P25 and the photocatalytic activity of α-FeOOH–TiO_2_. In the experiments with UV light and α-FeOOH–TiO_2_, mechanically activated, a 5.4 log-reduction of bacteria was achieved after 240 min of treatment. Using visible light the α-FeOOH–TiO_2_ and the TiO_2_-P25 showed a 3.1 and a 0.7 log-reductions at 240 min, respectively. The disinfection mechanism was studied by ROS detection and scavenger experiments, demonstrating that the main ROS produced in the disinfection process were superoxide radical anion, singlet oxygen and hydroxyl radical.

## Abstract 2

A photocatalytic mechanism for FeOOH–TiO_2_ composite is proposed under UV-Vis light, the FeOOH–TiO_2_ composite showed higher photocatalytic activity than TiO_2_-P25.

## Graphical abstract

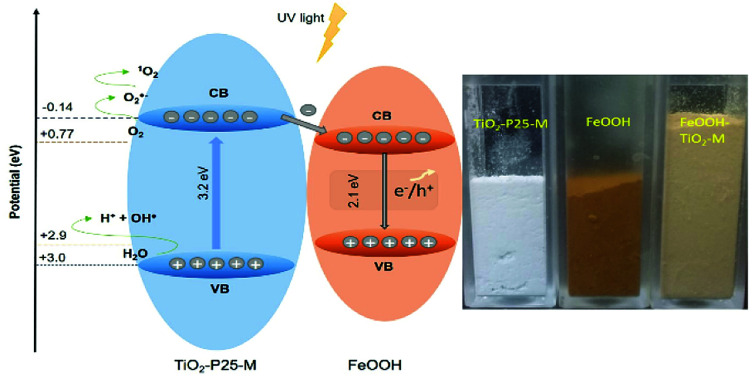

## 1. Introduction

In 2017 the WHO/UNICEF reported that 844 million people did not have a drinking water facility. Microbial contamination (*E. coli* or thermotolerant coliforms) of water is a worldwide concern.^1^ It was estimated that 477 291 children from 0 to 4 years died in 2016 due to diarrhea.^2^ In developing regions, the use of solar disinfection is one of the most reliable treatments for water disinfection. However, this technique can be improved using non-toxic and earth-abundant oxides with photocatalytic activity. TiO_2_ is a semiconductor employed in disinfection processes.^3–9^ The disinfection mechanism involves the production of reactive oxygen species (ROS), under UV light, through oxidation and reduction reactions by holes (h^+^) and electrons (e^−^) in the valence and conduction bands of TiO_2_, respectively. The main ROS produced are superoxide radical anion (O_2_˙^−^), singlet oxygen (^1^O_2_), hydroxyl radical (·OH) and hydrogen peroxide (H_2_O_2_). Investigations related to TiO_2_ have been carried out to decrease the recombination rate of e^−^–h^+^ pairs and increase its absorption to the visible range. Goethite (α-FeOOH) is an abundant iron oxyhydroxide,^10^ with a band gap value of 2.1 eV.^11^ Few works have been reported using FeOOH–TiO_2_ composites for *E. coli* disinfection.^12,13^ In these works, the route used to obtain the composites was the hydrothermal method. Chowdhury and Mpongwana^12^ studied the FeOOH–TiO_2_ composite, FeOOH was akaganeite (β-FeOOH), for *E. coli* disinfection in presence of H_2_O_2_ as electron acceptor; Mangayayam *et al.* studied the disinfection efficiency of *E. coli* using Ag–TiO_2_–FeOx (mainly goethite 37.3%) nanotubes.^13^ Goethite–titania composites can show enhanced photocatalytic activity due to the related studies reporting the increase of TiO_2_ light absorption attributed to Fe doping or iron oxides addition, and the photo-Fenton and photocatalytic activity of goethite.^11,14–16^ Additionally, the mechanical activation, through ball milling, has shown to create defects in materials, which contributes to improving its catalytic properties.^17,18^

The aim of this work was to investigate the photocatalytic mechanism of α-FeOOH–TiO_2_ composites in the disinfection of *E. coli* under UV and visible light irradiation. The composites were synthesized by co-precipitation and mechanical milling. The photocatalytic mechanism of the composite was studied by means of ROS detection and scavengers addition.

## 2. Materials and methods

### 2.1 Synthesis of goethite (α-FeOOH)

Goethite was synthesized by co-precipitation method (Fig. S1†). A solution of NaOH (Fermont, Mexico) 10 M was dripped in 200 mL of FeSO_4_·7H_2_O (Jalmek, Mexico) 0.04 M until pH 13 and kept under constant stirring with air sparging, after 4 h a yellow dark precipitate was obtained. The precipitate was washed with deionized water several times and centrifuged at 11 000 rpm for 10 min (Thermo Scientific, Sorvall ST-16). Finally, it was dried at 90 °C for 24 h.

### 2.2 Synthesis of FeOOH–TiO_2_ composites

The syntheses of the composites were carried out by two techniques, the first labeled *in situ* (Fig. S2†), where TiO_2_-P25 (Aeroxide, Evonik, Degussa Corporation) was added to the solution after 4 h of goethite synthesis and kept for 1 h under constant stirring with air sparging. The second technique was by mechanical activation of TiO_2_-P25 and goethite previously synthesized (Fig. S3†), using a planetary mill (Retsch PM 100) with a ratio of 10 : 1 (balls : load) at 450 rpm for 1 h, with ethanol as dispersing agent, and zirconia container and balls. The powders were dried at 80 °C for 12 h. For both syntheses, the stoichiometric ratios used were 1 : 1, 1 : 3 and 3 : 1 for FeOOH and TiO_2_-P25, respectively.

### 2.3 Characterization

The materials were characterized by X-ray powder diffraction (XRD, Panalytical, Empyrean) with Cu Kα radiation at 40 kV and 30 mA. The morphology was investigated by scanning electron microscopy (FE-SEM JEOL JSM-7041F and FEI Nova Nanosem 200). The UV-visible absorption was analyzed by diffuse reflectance in a UV-visible spectrometer (AvaSpec-2048L, Avantes).

### 2.4 Photocatalytic disinfection of *E. coli*

#### 2.4.1. Reactor configuration

The photochemical reactor employed for the disinfection experiments consisted of a borosilicate glass beaker of 0.2 L surrounded by a water-jacket and ports for sampling and air sparging (Fig. S4†). The lamp was inserted into a borosilicate tube located inside the reactor. The borosilicate tube served as a cut-off filter for UVC light (*λ* < 285 nm). For UV experiments the suspension was irradiated using a Hg–Ne lamp (Pen-ray®, UVP). The visible assays were carried out with a 1.8 W low-intensity LED lamp, with an emission range from 420 nm to 620 nm, as shown in Fig. S5.† Potassium ferrioxalate (1.43 × 10^−9^ einstein per cm^2^ per s) and Reinecke's salt (4.47 × 10^−8^ einstein per cm^2^ per s) actinometries were performed for UV and LED lamps, respectively.^19^

#### 2.4.2. *E. coli* inoculum and solution

*E. coli* K-12 (ATCC 25404) was incubated aerobically at 37 °C in Luria Bertani (LB) broth (Sigma Aldrich). After 18 h of incubation, the bacteria were collected giving a concentration of 10^8^ CFU mL^−1^. The bacteria suspension was centrifuged at 4500 rpm for 10 min, washed three times with phosphate buffer pH 7. Finally, the bacteria were diluted to 10^6^ CFU mL^−1^ in saline solution.

#### 2.4.3. Disinfection experiments

The experiments were performed using 0.2 L of *E. coli* (10^6^ CFU mL^−1^), UV or visible light irradiation, air sparging and kept at 20 °C ± 2. The disinfection of *E. coli* was evaluated employing TiO_2_-P25 (with and without mechanical activation), goethite and FeOOH–TiO_2_ composites obtained by *in situ* and mechanical activation. First, disinfection experiments using TiO_2_-P25, as reference, at different concentrations (72, 150, 300 and 500 mg L^−1^) were performed in order to select the concentration with higher disinfection efficiency. The concentration of 300 mg L^−1^ was selected to compare the efficiency of the materials described in Table 1.

**Table 1:** Materials used for the UV or visible disinfection experiments

Identification	Description
P25	TiO_2_-P25 without treatment
P25-M	TiO_2_-P25 milled at 400 rpm for 1 h
G	Goethite without treatment
1 : 3-I	Molar ratio 1 : 3 goethite : TiO_2_-P25, *in situ*
1 : 3-M	Molar ratio 1 : 3 goethite : TiO_2_-P25, milled at 450 rpm for 1 h
1 : 1-I	Molar ratio 1 : 1 goethite : TiO_2_-P25, *in situ*
1 : 1-M	Molar ratio 1 : 1 goethite : TiO_2_-P25, milled at 450 rpm for 1 h
3 : 1-I	Molar ratio 3 : 1 goethite : TiO_2_-P25, *in situ*
3 : 1-M	Molar ratio 3 : 1 goethite : TiO_2_-P25, milled at 450 rpm for 1 h

Aliquots were collected at different times for 300 min and bacteria concentration was determined using the standard plated counting method on LB agar by triplicate. The detection limit was 2 CFU mL^−1^ and was achieved inoculating 500 μL of sample. The plates were incubated for 24 h at 37 °C. Control experiments were made without material to evaluate the photolysis and with material in the dark.

### 2.5 ROS detection

#### 2.5.1. Singlet oxygen (^1^O_2_)

*p*-Nitrosodimethylaniline (RNO) and imidazole has been reported for singlet oxygen detection.^20–22^ TiO_2_-P25, TiO_2_-P25-M, G, 1 : 3-M, 1 : 1-M, 3 : 1-M, 1 : 3-I, 1 : 1-I or 3 : 1-I at 300 mg L^−1^ were suspended in a solution containing RNO (Sigma Aldrich) 45 μM and imidazole (Sigma Aldrich) 8 mM. The experiments were performed under UV or visible light. Aliquots were taken during 24 min and centrifuged at 12 000 rpm for 15 min. The RNO concentration was determined at 440 nm in a Varian Cary 50 UV-Vis spectrometer. Negative controls were made without material.

#### 2.5.2. Superoxide radical (O˙^−^_2_)

XTT sodium salt (C_22_H_16_N_7_NaO_13_S_2_) is reduced by O˙^−^_2_ giving the product XTT-formazan.^23^ In 100 mL of XTT 100 μM (Sigma Aldrich), 300 mg L^−1^ of the corresponding material was placed and kept under magnetic stirring. The solutions were prepared in deionized water. The experiments were performed under UV or visible light. Every 2–3 min samples were collected and centrifuged. The concentration of XTT-formazan was determined at 470 nm. Controls were performed without material.

#### 2.5.3. Hydroxyl radical (OH˙)

In 100 mL of RNO (0.017 mM), 300 mg L^−1^ of material was placed. The experiments were performed under UV or visible light. Each 2–3 min aliquots were taken, centrifuged and read at 440 nm.^24,25^ Controls were performed without material.

#### 2.5.4. Hydrogen peroxide (H_2_O_2_)

Equal volumes of acidified titanium(iv) oxysulfate (Sigma Aldrich) 280 mM and sample, material in saline solution previously irradiated with UV or visible light, were mixed and its absorbance was read at 410 nm.^26^

### 2.6 Scavenger addition

Disinfection experiments were performed with different scavengers. *tert*-Butanol (TBA, 10 mM) was used as hydroxyl radical scavenger; KI (10 mM) quenched surface holes and surface bounded ·OH. The reduction pathway was inhibited by introducing nitrogen into the solution.

### 2.7 Addition of H_2_O_2_ as electron acceptor

*E. coli* disinfection experiments in presence of 1 : 3-M (300 mg L^−1^) and H_2_O_2_ (10 mg L^−1^), as electron acceptor, under visible light were performed.

## 3. Results and discussion

**Fig. 1:**
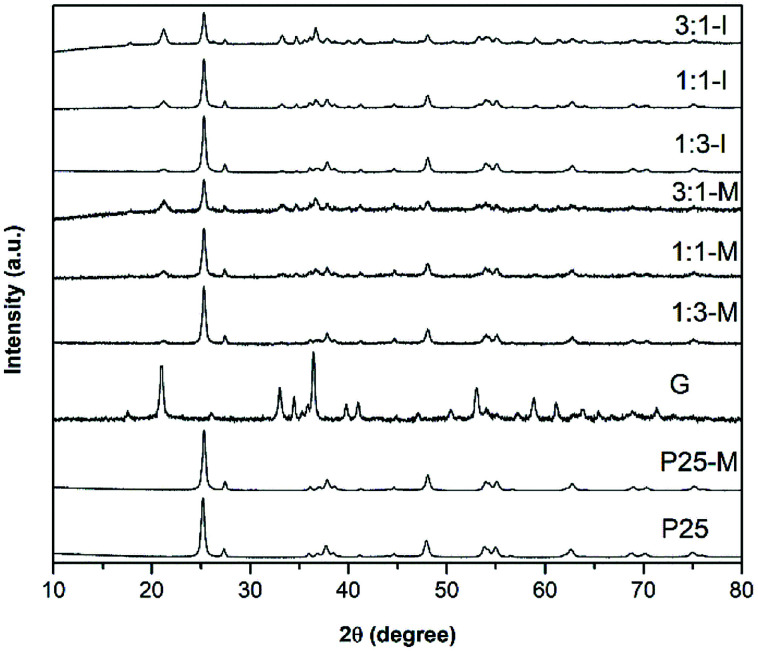
XRD patterns in relative intensities of P25, P25-M, G, 1 : 3-M, 1 : 1-M, 3 : 1-M, 1 : 3-I, 1 : 1-I and 3 : 1-I composites.

Fig. 1 shows the XRD patterns of P25, P25-M, G, 1 : 3-M, 1 : 1-M, 3 : 1-M, 1 : 3-I, 1 : 1-I and 3 : 1-I. The phases detected correspond to the indexed PDF crystallographic cards 01-075-2545 (anatase) and 01-080-2533 (rutile) for P25 and P25-M, and 98-007-1808 for goethite. The crystallite size of P25 and P25-M was calculated using the Scherrer equation (eqn (1)). The results showed a crystallite size of 19.4 nm for P25-M and 18.8 nm for P25.

1 *D* = *Kλ*/(*β* cos *θ*)

**Fig. 2:**
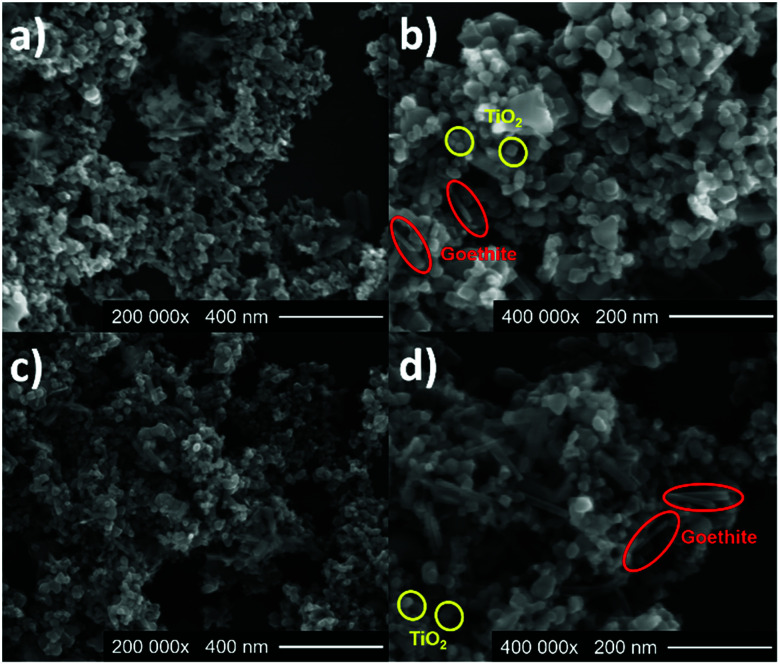
SEM micrographs of 1 : 3-I (a and b) and 1 : 1-M (c and d).

SEM characterization was carried out to investigate the shape and size of the photocatalysts. Fig. 2a and b shows SEM images of 1 : 3-I with densely packed FeOOH rods with TiO_2_-P25 spherical particles with a longitude of 126.5 ± 29 nm and 22.1 ± 2 nm, respectively. Fig. 2c and d corresponds to the composite 1 : 3-M, the FeOOH and the TiO_2_-P25 show the same morphology that 1 : 3-I and longitudes of 86.1 ± 18 nm and 19.4 ± 6 nm, respectively. FE-SEM images of P25, P25-M and G are in Fig. S6.†

**Fig. 3:**
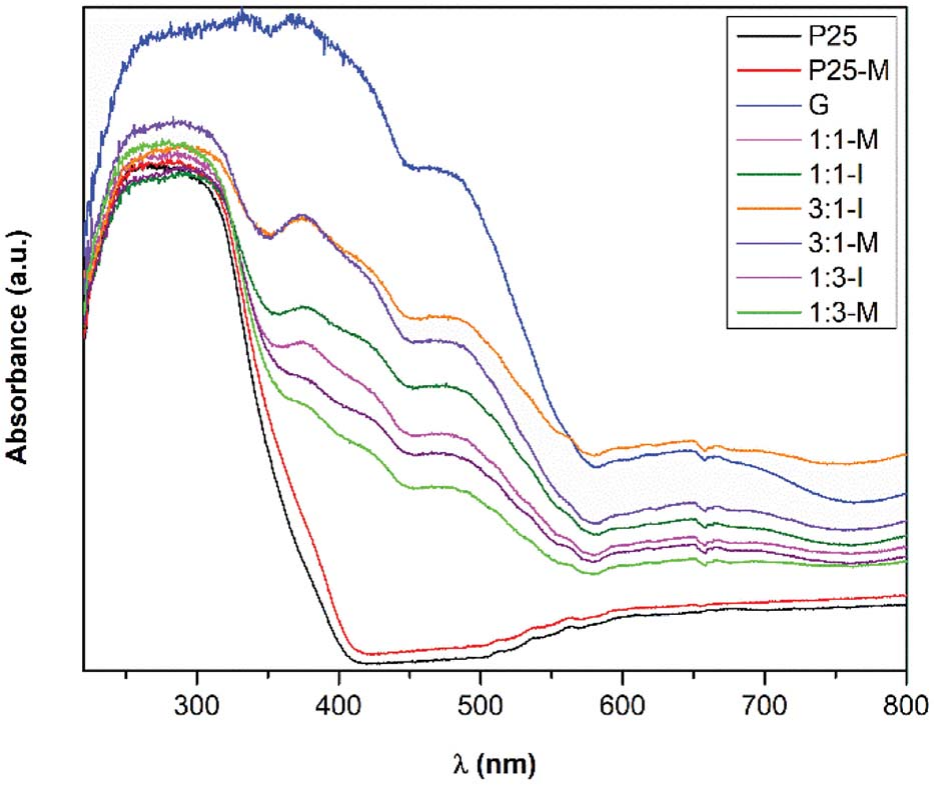
UV-Vis absorption spectra of P25, P25-M, G, 1 : 3-M, 1 : 1-M, 3 : 1-M, 1 : 3-I, 1 : 1-I and 3 : 1-I composites.

Fig. 3 displays the UV-Vis absorption spectra of P25, P25-M, G, 1 : 3-M, 1 : 1-M, 3 : 1-M, 1 : 3-I, 1 : 1-I and 3 : 1-I. For P25 and P25-M, a strong absorption in the UV until 410 and 415 nm, respectively, is observed. The diffuse reflectance spectra were used to estimate the bandgaps of all samples using the Kubelka–Munk function (eqn (2), Fig. S7†). The band gap values were 3.3, 3.2, 2.13, 2.86, 2.85, 2.83, 2.81, 2.76 and 2.73 eV for P25, P25-M, G, 1 : 3-M, 1 : 1-M, 3 : 1-M, 1 : 3-I, 1 : 1-I and 3 : 1-I composites, respectively. The mechanical activation reduced the band gap value in P25 only.

2 *K*/*S* = FKM(*R*) = (1 − *R*)^2^/2*R*

According to Dannangoda *et al.*, the reduction in the band gap value after mechanical activation can be related with the change in bond angles and lengths in the crystal structure by the impact during the milling process.^27^

### 3.1 Photocatalytic disinfection with UV light

**Fig. 4:**
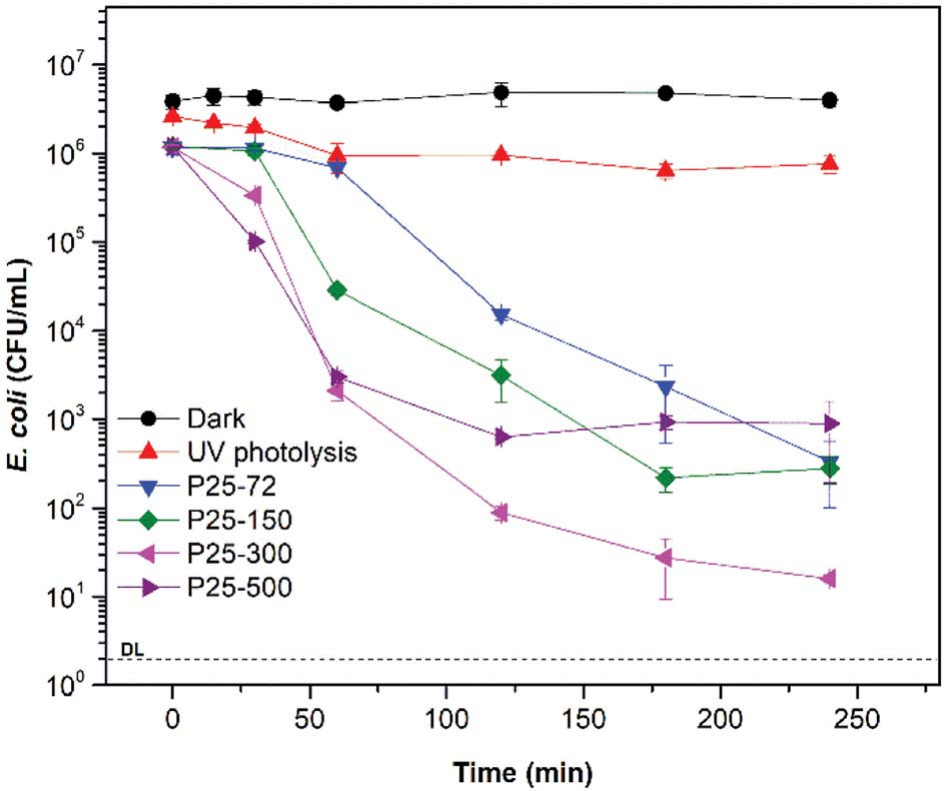
Bacterial disinfection with TiO_2_-P25 at 72 mg L^−1^, 150 mg L^−1^, 300 mg L^−1^ and 500 mg L^−1^ under UV light.

In Fig. 4 the disinfection plot using TiO_2_-P25 at 72, 150, 300 and 500 mg L^−1^ with UV light is shown. TiO_2_-P25 at 300 mg L^−1^ reached a 4.5 log-reduction after 240 min of irradiation. With 72 and 150 mg L^−1^ of TiO_2_-P25 log-reductions of 3.7 and 3.8 at 240 min were achieved, respectively. TiO_2_-P25 at 500 mg L^−1^ gave the lower disinfection efficiency. The *E. coli* concentration remained constant during the 300 min in the dark. In the photolytic experiment, a 0.5 log-reduction at 240 min was observed. The following experiments with goethite and the composites under UV or visible irradiation were performed at 300 mg L^−1^.

**Fig. 5:**
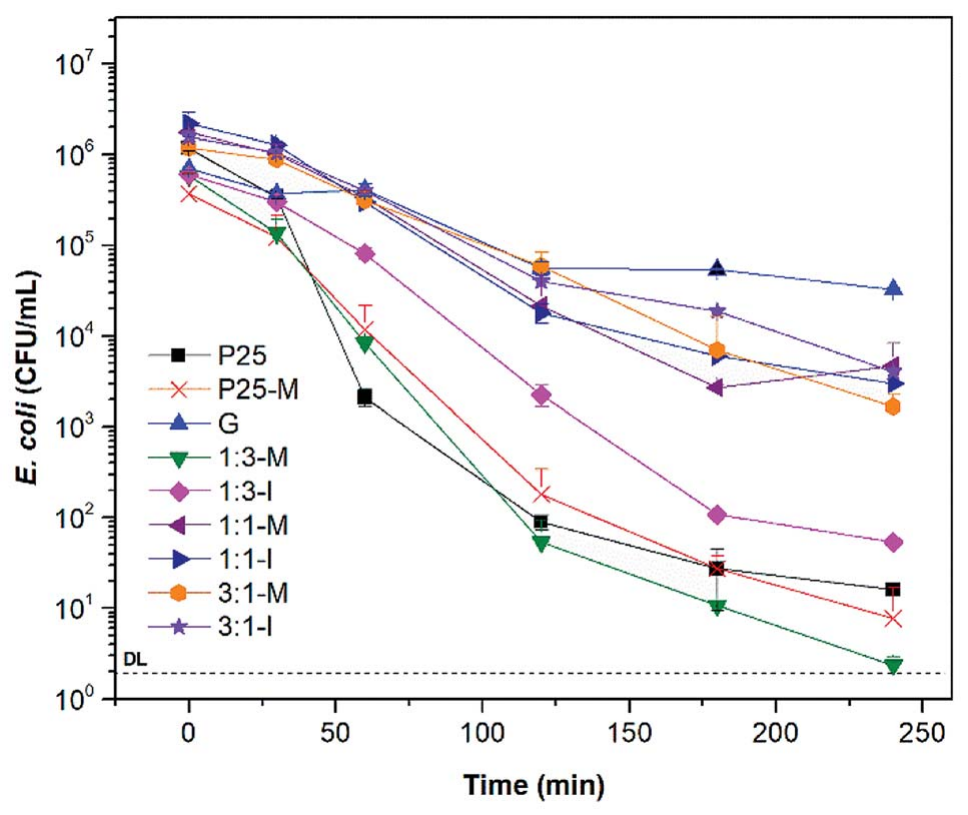
Bacterial disinfection with P25, P25-M, G and FeOOH–TiO_2_ composites under UV light at 300 mg L^−1^.

TiO_2_–FeOOH (1 : 3-M) is more photocatalytic active under UV light than TiO_2_-P25. With 300 mg L^−1^ of TiO_2_–FeOOH (1 : 3-M) a disinfection efficiency of 5.1 log-reduction was reached at 240 min (Fig. 5). TiO_2_-P25-M showed a slightly better disinfection efficiency than TiO_2_-P25 with a 4.7 log-reduction at 240 min. In general, it is possible to observe that the composite with FeOOH at a low molar ratio and with mechanical activation increases the disinfection efficiency, the later can be due to the defects created during the mechanical activation.^17,18^ Ruales *et al.* reported that goethite acts as an efficient photocatalyst in absence of H_2_O_2_, in our case we only could appreciate a 1.34 log-reduction at 240 min.^11^ The dark controls of *E. coli* disinfection with the different photocatalysts are in Fig. S8.†

### 3.2 Disinfection with visible light

**Fig. 6:**
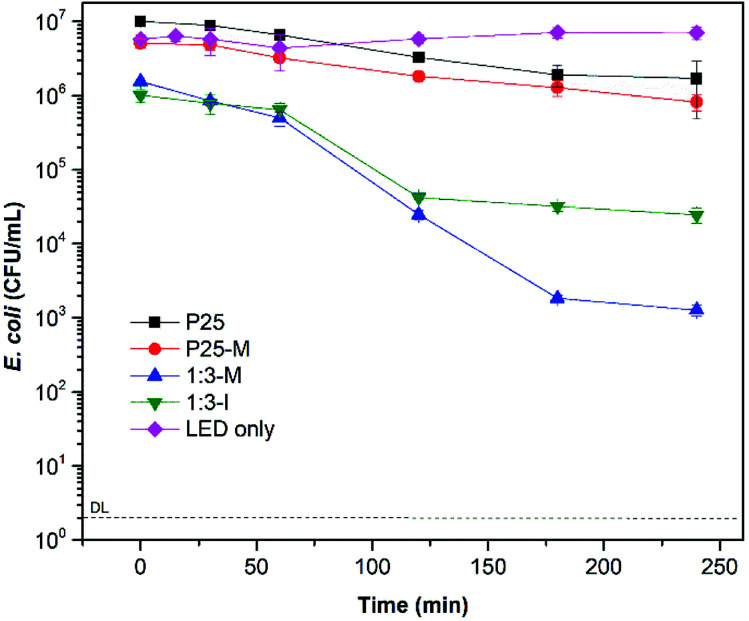
Bacterial disinfection with P25, P25-M and FeOOH–TiO_2_ 1 : 3 composites under visible light at 300 mg L^−1^.

Fig. 6 shows the disinfection kinetics of *E. coli* under visible light with P25, P25-M, 1 : 3-M and 1 : 3-I. With 1 : 3-M a 3.1 log-reduction after 240 min of treatment was achieved. In the case of P25, P25-M and 1 : 3-I showed a 0.7, 0.8 and 1.5 log-reduction at the same time, respectively. The results confirm that 1 : 3-M shows higher photoactivity and absorption range than TiO_2_-P25.

The fitting tool GInaFit, version 1.7 was employed to analyze the disinfection kinetic curves.^28^ The results are shown in Table 2. The shoulder indicates the time before the bacteria concentration begins to diminish, and the tail the moment in which an additional reduction is not achieved, probably due to bacteria resistance or a protective effect of the residual cell components to the still viable bacteria.

**Table 2:** Effect of P25, P25-M, G and FeOOH–TiO_2_ composites on *E. coli* disinfection under UV or visible light: kinetic parameters found using the Gina-Fit tool with the log linear + shoulder + tail model

Material (mg L^−1^)	Parameter				
	Material (mg L^−1^)	SI (shoulder length, min)	*K*_max_ (min^−1^)	log *N*_res_ (residual bacterial concentration)	*N*_o_ (initial bacterial concentration)
UV light	P25 (72)^a^	46.72 ± 22.08	0.05 ± 0.01	2.43 ± 0.31	6.15 ± 0.43
	P25 (150)	14.00 ± 30.37	0.06 ± 0.02	2.36 ± 0.34	6.17 ± 0.43
	P25 (300)	25.00 ± 10.73	0.18 ± 0.05	1.53 ± 0.22	6.07 ± 0.38
	P25 (500)	10.93 ± 1.01	0.13 ± 0.01	2.77 ± 0.01	6.08 ± 0.02
	P25-M (300)	15.25 ± 4.05	0.07 ± 0.01	0.91 ± 0.06	5.58 ± 0.07
	G (300)	70.11 ± 23.03	0.07 ± 0.04	4.62 ± 0.12	5.74 ± 0.14
	1 : 3-M (300)	13.03 ± 14.79	0.09 ± 0.01	0.68 ± 0.24	5.78 ± 0.32
	1 : 3-I (300)^a^	29.10 ± 1.11	0.06 ± 0.01	1.72 ± 0.02	5.77 ± 0.01
	1 : 1-M (300)^a^	33.43 ± 8.11	0.05 ± 0.00	3.41 ± 0.07	6.24 ± 0.08
	1 : 1-I (300)	23.61 ± 12.73	0.05 ± 0.01	3.58 ± 0.11	6.37 ± 0.12
	3 : 1-M (300)^a^	38.58 ± 17.18	0.04 ± 0.01	3.68 ± 0.14	6.08 ± 0.11
	3 : 1-I (300)^a^	9.92 ± 38.87	0.03 ± 0.01	3.44 ± 0.51	6.25 ± 0.20
Visible light	P25 (300)^a^	67.79 ± 5.24	0.03 ± 0.00	6.21 ± 0.02	7.00 ± 0.01
	P25-M (300)^a^	46.02 ± 5.19	0.02 ± 0.01	5.77 ± 0.28	6.73 ± 0.05
	1 : 3-M (300)^a^	63.96 ± 6.14	0.06 ± 0.01	3.07 ± 0.07	6.14 ± 0.05
	1 : 3-I (300)	68.98 ± 7.84	0.08 ± 0.01	4.44 ± 0.05	5.96 ± 0.06

a No fits on tail model.

### 3.3 ROS production

Eqn (3) shows the reaction between the RNO and the hydroxyl radical, which produces the bleaching of the RNO.

3 RNO + HO˙ → RNO·OH

**Fig. 7:**
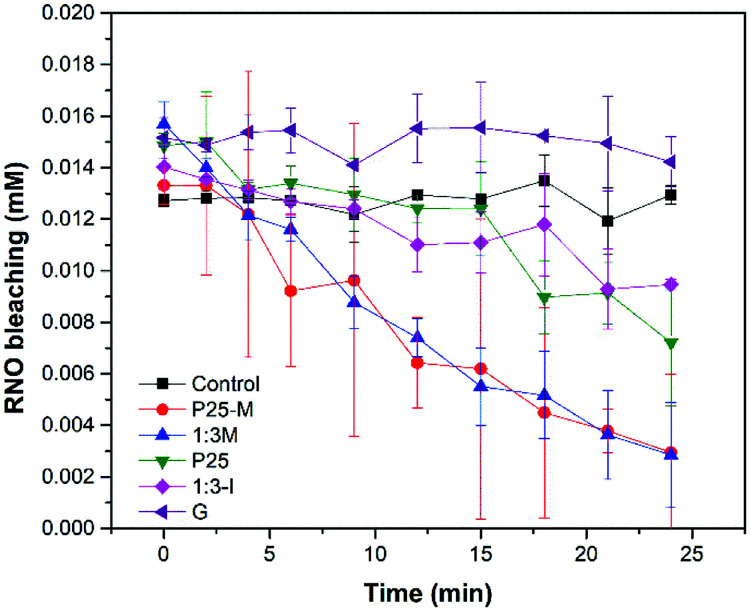
Hydroxyl radical detection under UV light irradiation.

In Fig. 7 the materials P25, P25-M, 1 : 3-M and 1 : 3-I produced ·OH under UV irradiation. However, it was not possible to detect ·OH with goethite. P25-M and 1 : 3-M produced almost the same quantity of ·OH. The reaction rate coefficients (*k*) for the control, P25, P25-M, G, 1 : 3-M and 1 : 3-I are 5 × 10^−6^, 0.0119, 0.0281, 0.0005, 0.0306 and 0.007, respectively. The data were analyzed by a factorial design and the interaction graphs for each ROS test are in the ESI (Fig. S9–S11†).

**Fig. 8:**
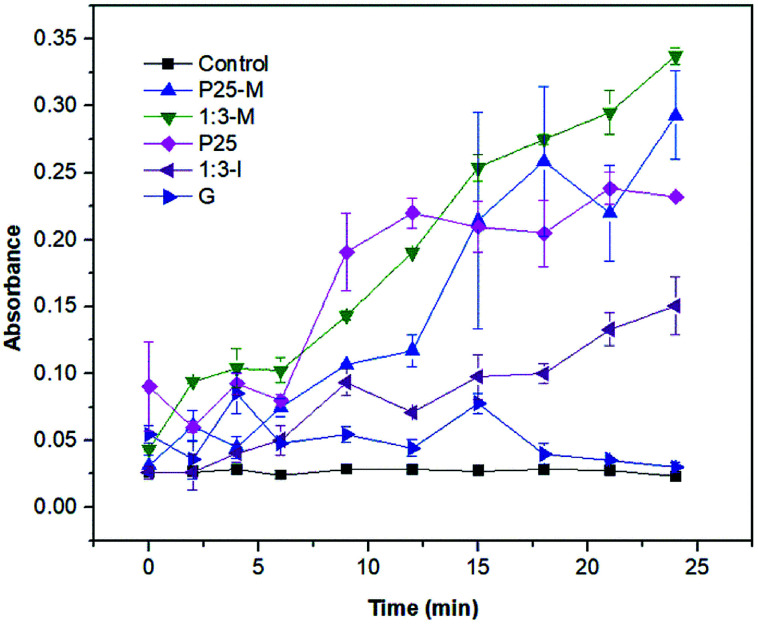
Superoxide detection under UV light irradiation.

For superoxide detection (Fig. 8) the XTT reduction by superoxide to XTT-formazan was monitored. The 1 : 3-M composite exhibited more superoxide production than P25-M. Thus, the interaction between FeOOH and TiO_2_-P25-M increased the disinfection photocatalytic efficiency compared to α-FeOOH or TiO_2_-P25. The reaction rate coefficients (*k*) for the control, P25, P25-M, G, 1 : 3-M and 1 : 3-I are 0.0006, 0.0241, 0.0394, 0.0084, 0.0327 and 0.0318, respectively.

**Fig. 9:**
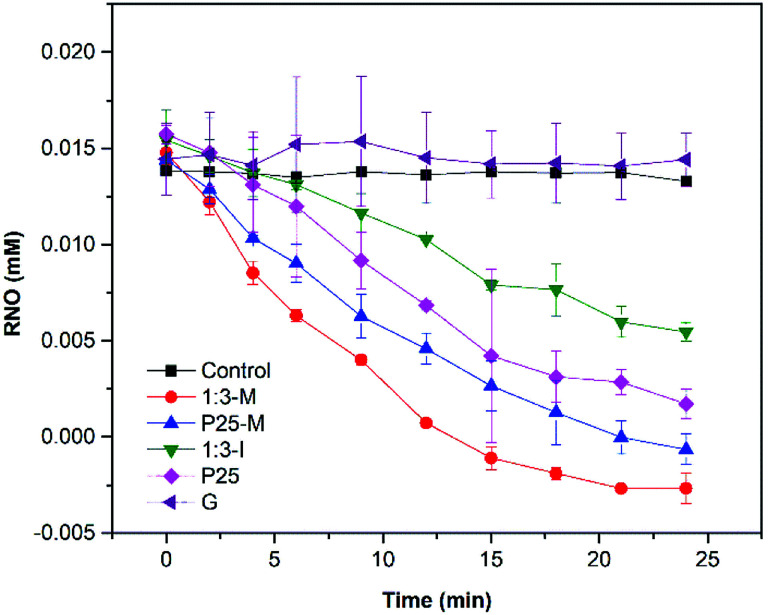
Singlet oxygen detection under UV light irradiation.

According to Kralji and El Mohsni, the reaction of imidazole with ^1^O_2_ generates an intermediate that reacts with RNO.^20^ The singlet oxygen detection (Fig. 9) showed that only 1 : 3-M, P25-M, P25 and 1 : 3-I produced ^1^O_2_ under UV irradiation. The reaction rate coefficients (*k*) for the control, P25, P25-M, G, 1 : 3-M and 1 : 3-I are 0.0003, 0.0411, 0.0561, 0.0006, 0.101 and 0.0197, respectively.

The tests between 1 : 3-M and Ti(iv) ions showed no significant H_2_O_2_ formation during 24 min under UV or visible light. The same result was observed using different material loadings (data not shown).

ROS detection under visible irradiation was negative, probably due to the low detection limit of spectroscopic probes. The same behavior was observed in previous work,^29^ where hydroxyl radical was detected only under UV irradiation.

### 3.4 Scavenger study under visible irradiation

The addition of *tert*-butanol (TBA) showed a reduction in the disinfection efficiency compared to 1 : 3-M without TBA (Fig. S12†), suggesting that the disinfection is mediated by hydroxyl radicals in the bulk. When KI was used, as a surface hole and hydroxyl radical scavenger, the disinfection efficiency increased considerably. Probably due to the disinfectant action of iodine formed after oxidation of iodide, such behavior was also observed in previous work.^29^ Under anoxic conditions the disinfection also decreased, pointing out that the oxygen reduction pathway plays an important role in ROS production. It is necessary to emphasize that the study with scavengers must be interpreted with caution since they can be involved in side reactions, mainly in disinfection processes.

In the experiment of 1 : 3-M with visible light and H_2_O_2_ (Fig. S4†) significant *E. coli* disinfection was observed compared to 1 : 3-M under UV or visible irradiation without H_2_O_2_ (Fig. 5 and 6). Hydrogen peroxide also showed good disinfection efficiency at the concentration tested. These results follow those observed by Ruales *et al.* with goethite and peroxide.^11^

The eqn (4) and (5) were used to calculate the conduction band (CB) and the valence band (VB) potentials of P25-M and FeOOH.

4 *E*_VB_ = *X* − *E*_e_ + 0.5*E*_g_

5 *E*_CB_ = *E*_VB_ − *E*_g_

where *E*_VB_ is the valence band edge potential, *X* is the electronegativity of the semiconductor, for P25-M and FeOOH is 5.9 and 6.3 eV, respectively, *E*_e_ ≈ 4.5 eV, and *E*_g_ is the energy gap of the semiconductor.^30–32^

**Fig. 10:**
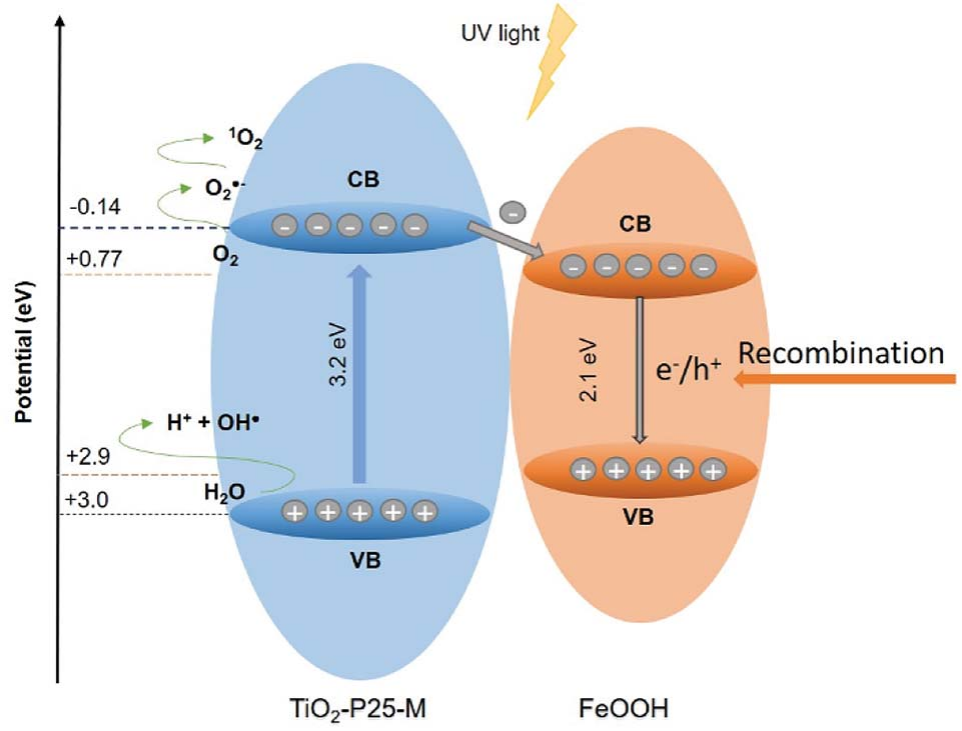
Proposed band alignment and photocatalytic mechanism for FeOOH–TiO_2_ composite.

Goethite did not show appreciable ROS production to determine its photoactivity; however, this does not mean that it does not contribute to the photocatalytic activity observed in the FeOOH–TiO_2_ composites. The band positions of TiO_2_-P25-M enable the generation of the ROS detected in this study. The heterojunction between these oxides improved the electron and hole mobility with a slight increase in the photoactivity of 1 : 3-M composite compared to TiO_2_-P25-M. In Fig. 10 the proposed photocatalytic mechanism, under UV light, between TiO_2_-P25-M and goethite shows the availability of holes and electrons in the VB and CB of TiO_2_-P25-M for oxidation and reduction reactions (eqn (6)–(8)). According to the ROS tests results, the goethite did not show production of ROS, this is attributed to rapid recombination of electron–hole pairs. The electrons in the CB of TiO_2_-P25-M that did not participate in the oxygen-reduction reactions migrate to the CB of goethite that acts as an electron capture site, which contributes to decrease the recombination in TiO_2_.^7^ Under visible light the composites showed photoactivity; however, a mechanism cannot be proposed because the ROS detection, under visible light, was negative, probably due to the low detection limit of the spectroscopic probes employed. Cruz-Ortiz *et al.* also studied the photoactivity of TiO_2_-P25, and according to the ROS study, the photocatalyst showed singlet oxygen production using the singlet oxygen sensor green (Invitrogen), a molecular probe that shows more sensitivity than the RNO-imidazole used in the present work.^29^ Also, the UV-visible absorption spectra of TiO_2_-P25 and TiO_2_-P25-M (Fig. 3) shows that these materials absorb in the visible region until 410 and 415 nm.

6 O_2_˙^−^ + h^+^ → ^1^O_2_

7 H_2_O + h^+^ → HO˙ + H^+^

8 O_2_ + e^−^ → O_2_˙^−^

## 4. Conclusions

FeOOH–TiO_2_ composite is a photocatalyst with increased disinfection activity than TiO_2_-P25. The better disinfection efficiency using UV or visible light was observed with FeOOH–TiO_2_ treated with mechanical milling. The ROS detected employing UV light were hydroxyl radical, superoxide radical anion and singlet oxygen. A reduction in the disinfection efficiency was detected when hole and hydroxyl radical scavengers, KI and *tert*-butanol, were added. Under N_2_ sparging the same effect was observed. A photocatalytic mechanism is proposed based on the band edge positions of TiO_2_ and FeOOH, and the ROS detected. Where the hydroxyl radical and singlet oxygen are generated after oxidation of H_2_O and superoxide radical in the valence band, respectively, and the superoxide is produced after oxygen reduction in the conduction band of the TiO_2_-P25.

## Conflicts of interest

There are no conflicts to declare.

## Supplementary Material

RA-009-C8RA08412B-s001
