# Supplementary material for: Goethite–titania composite: disinfection mechanism under UV and visible light
Source: RSC Adv. 2019 Jan 21;9(5):2792–8. doi: 10.1039/c8ra08412b (PMC9059932; doi:10.1039/c8ra08412b)
Supplement: RA-009-C8RA08412B-s001 [file RA-009-C8RA08412B-s001.pdf]

Electronic Supplementary Material (ESI) for RSC Advances.
This journal is © The Royal Society of Chemistry 2019

Supplementary information

Goethite-titania composite: disinfection mechanism under UV and visible light

Rosalina Lara-Rico,[a] Elia M. Múzquiz-Ramos, [a] Claudia M. López-Badillo, [a] Ulises M. García-Pérez, [b] and Brenda R. Cruz-Ortiz [*a]

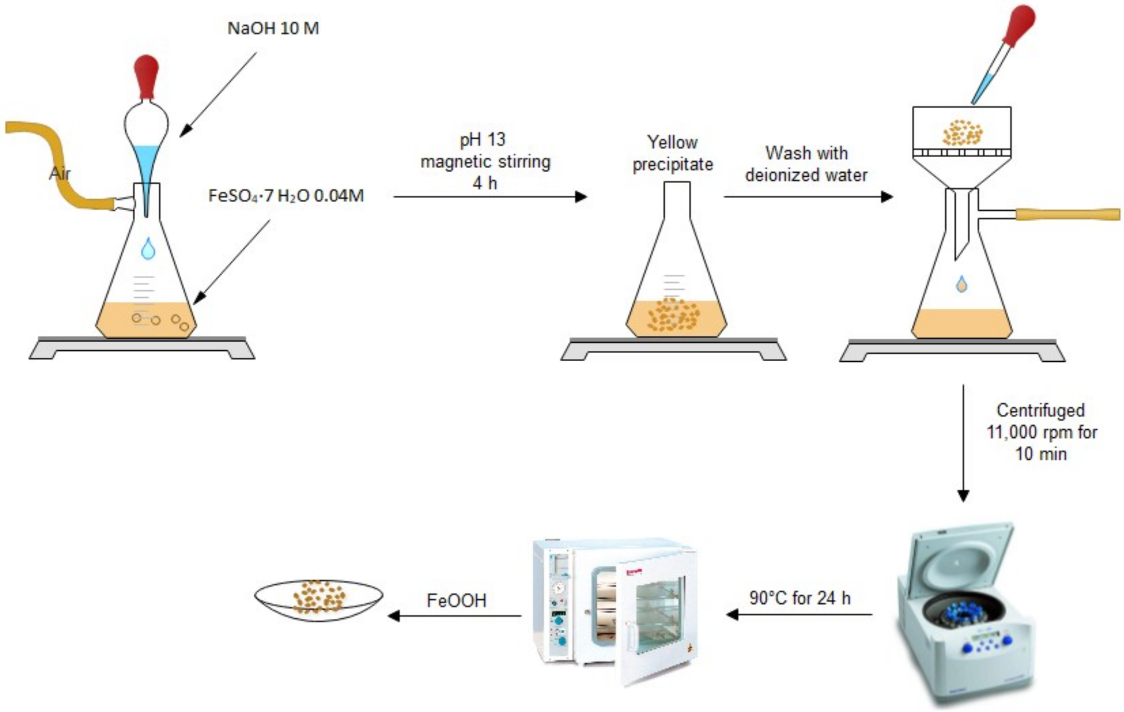

Fig. 1S Diagram of goethite synthesis.

Reactions of the synthesis route of goethite:

$$FeSO_4{\cdot}7H_2O_{(ac)} + 2\,NaOH_{(ac)} \rightarrow Na_2SO_{4(ac)} + Fe(OH)_{2\,(s)} + 7\,H_2O$$

$$2\,Fe(OH)_{2(s)} + \frac{1}{2}O_2 \rightarrow 2\,\alpha\,FeOOH_{(s)} + H_2O$$

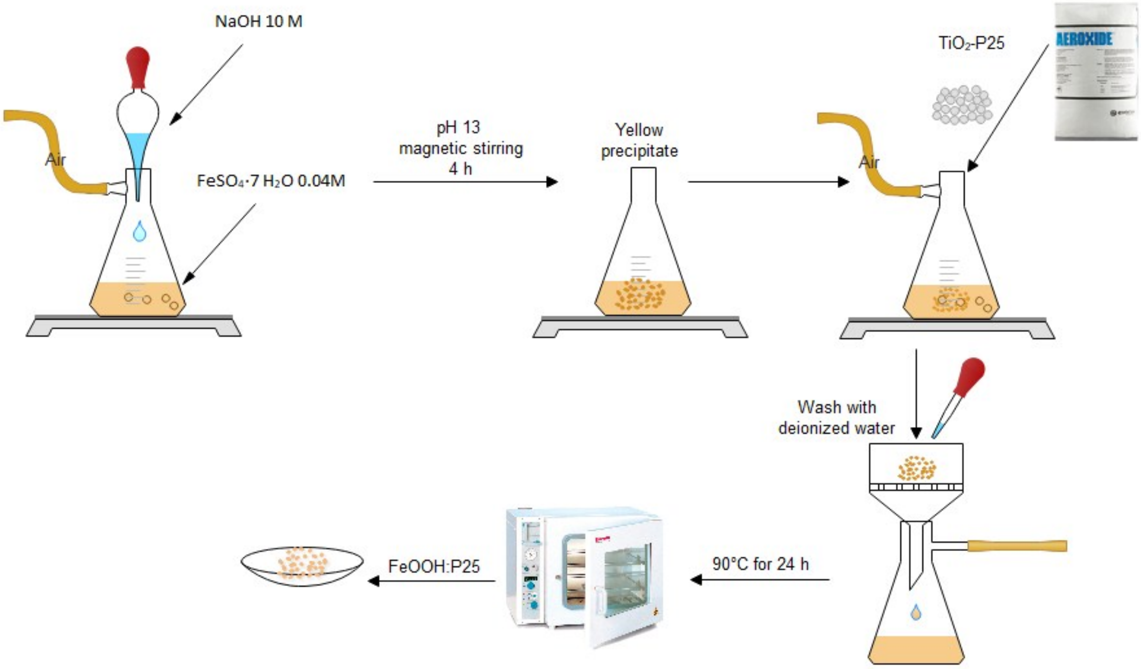

Fig. 2S Diagram of FeOOH:P25 composites by *in situ* method

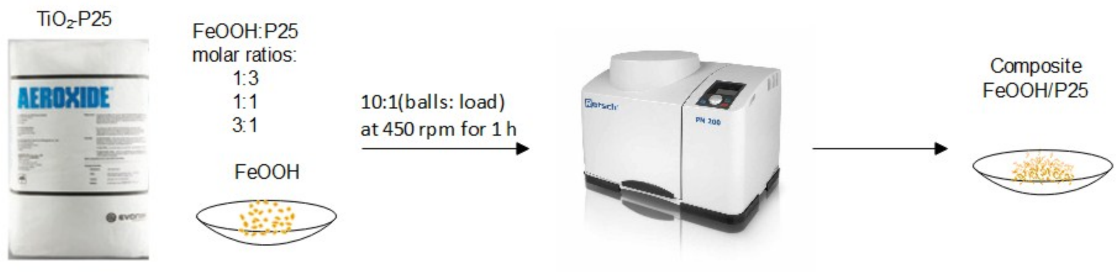

Fig. 3S Diagram of FeOOH:P25 composites synthesis by ball-milling.

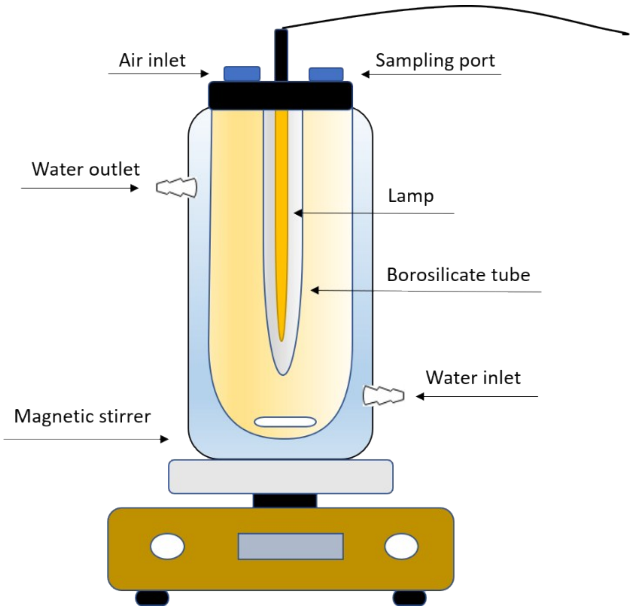

Fig. 4S Experimental reaction device diagram.

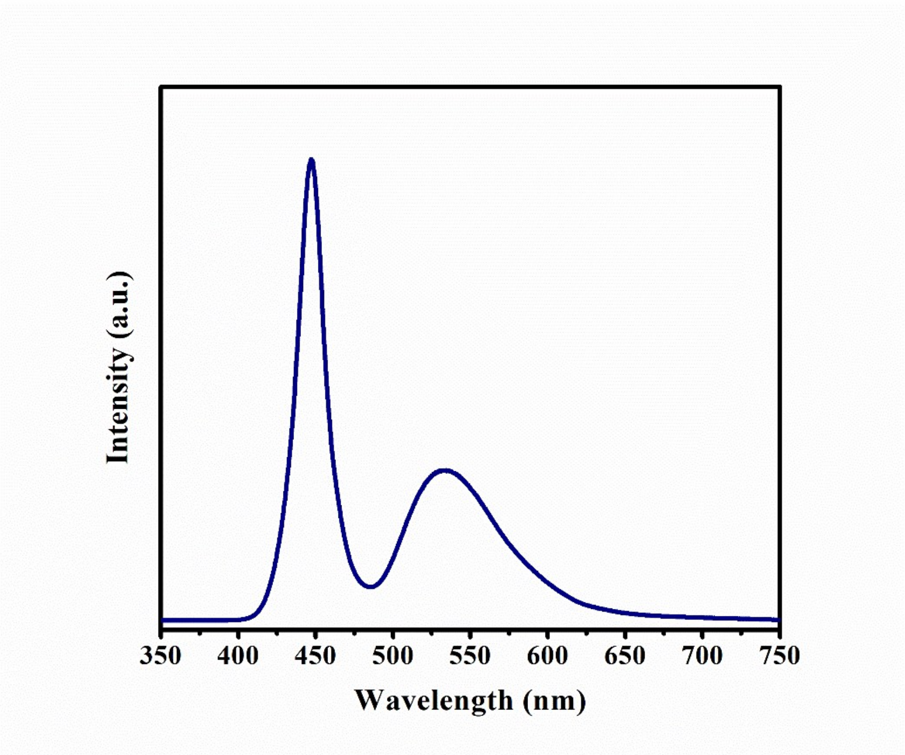

Fig 5S. Emission range of LED lamp.

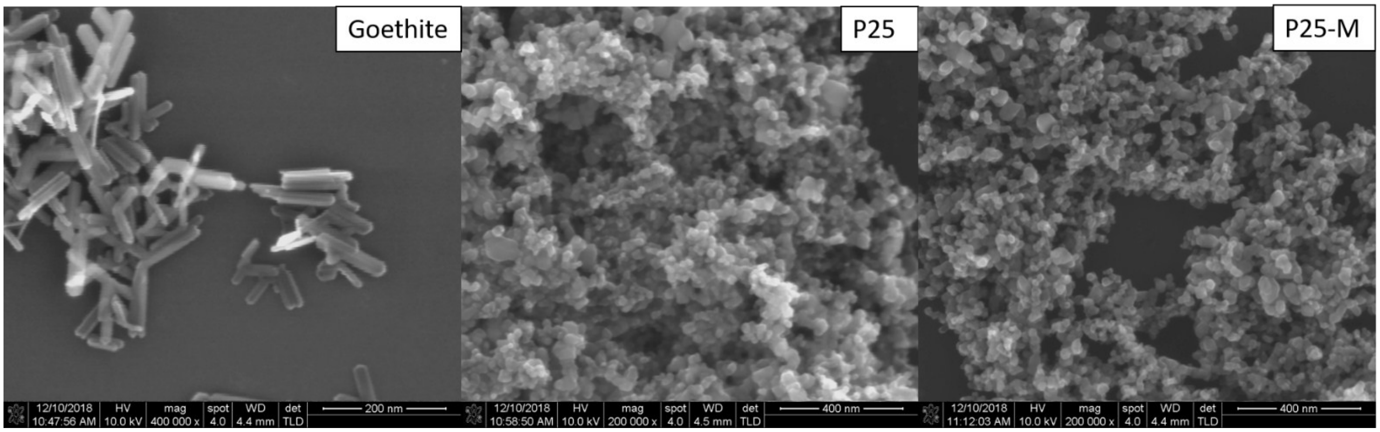

Fig. 6S FE-SEM images of goethite, P25 and P25-M.

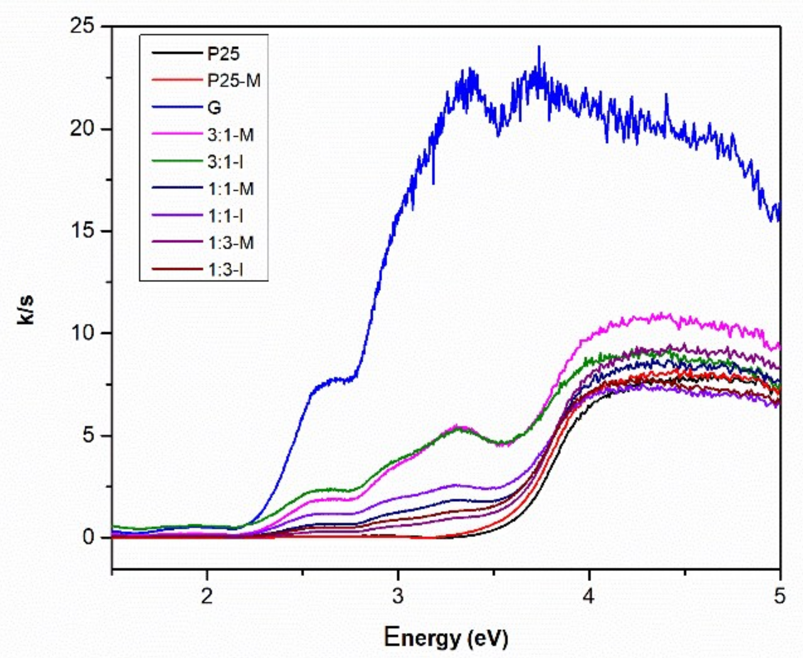

Fig. 7S. Determination of band gap values of P25, P25-M, G, 1:3-M, 1:1-M, 3:1-M, 1:3-I, 1:1-I and 3:1-I calculated using Kubelka-Munk function.

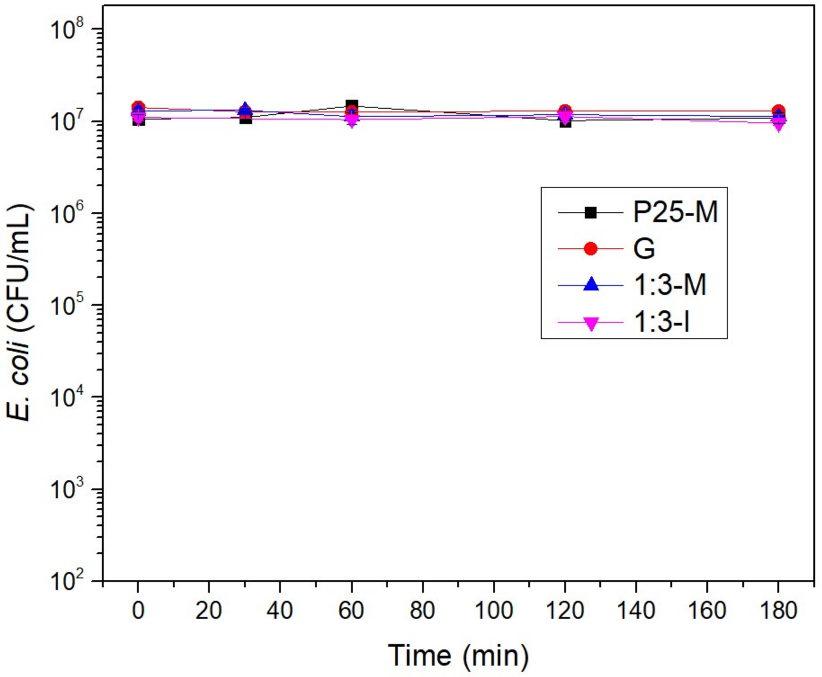

Fig. 8S *E. coli* disinfection with 300 mg/L of P25-M, G, 1:3-M or 1:3-I in dark conditions.

The interaction graphs of the analysis of the *OH at 0 and 24 min of the experiment shows that the control (1) and the goethite (4), at the end of the experiment, do not have significant difference with the zero time. The P25 and 1:3-I show similar difference in *OH production, and the samples with higher difference or higher *OH production are the P25-M and 1:3-M.

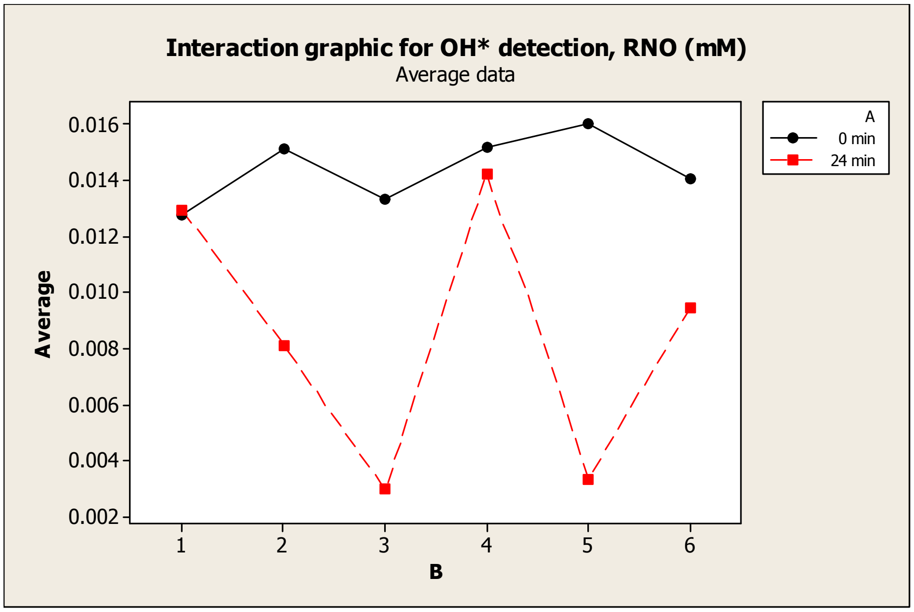

The interaction graphs of the analysis of O_2^{*-} at 0 and 24 min of the experiment shows that the control (1) and the goethite (4), at the end of the experiment, do not have significant difference with the zero time. The 1:3-I, P25, P25-M and 1:3-M show increasing *OH production.

Fig. 9S Interaction graph for *OH radical detection test. 1=control, 2=P25, 3=P25-M, 4=G, 5=1:3-M y 6=1:3-I.

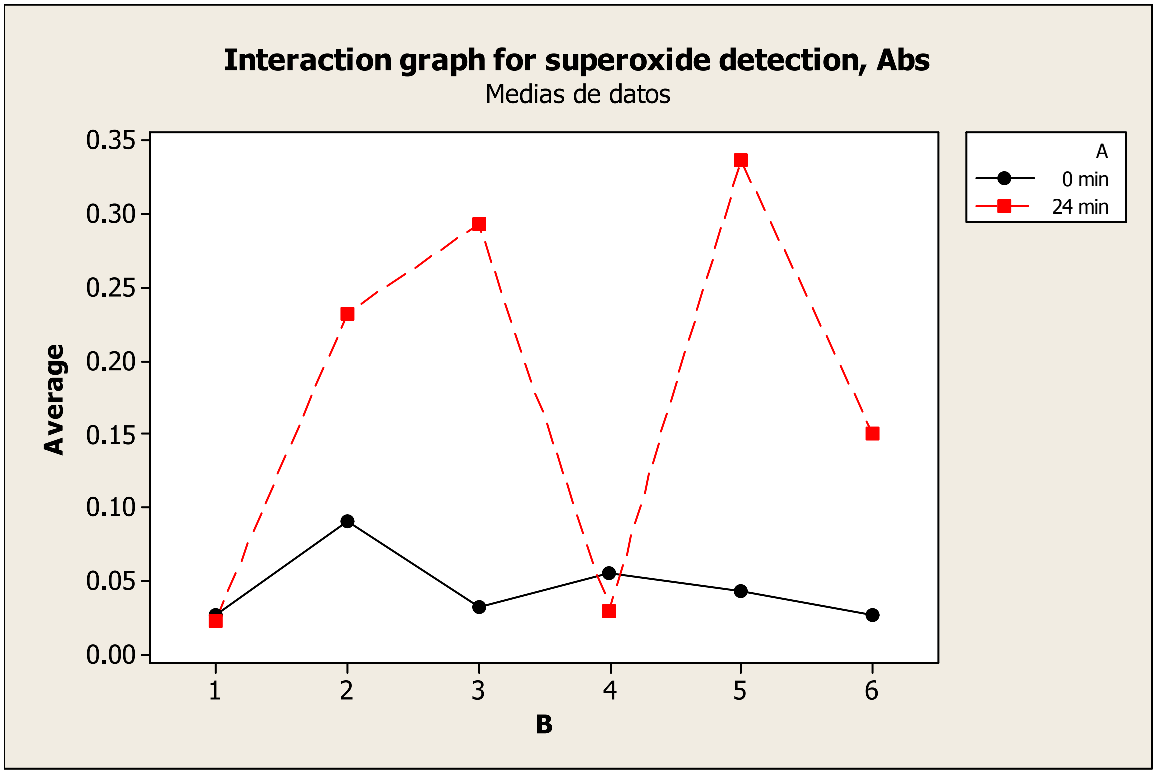

Fig. 10S Interaction graph for superoxide radical detection test. 1=control, 2=P25, 3=P25-M, 4=G, 5=1:3-M y 6=1:3-I.

The interaction graphs of the analysis of $^1O_2$ at 0 and 24 min of the experiment shows that the control (1) and the goethite (4), at the end of the experiment, do not have significant difference with the zero time. The 1:3-I, P25, P25-M and 1:3-M show increasing $^1O_2$ production.

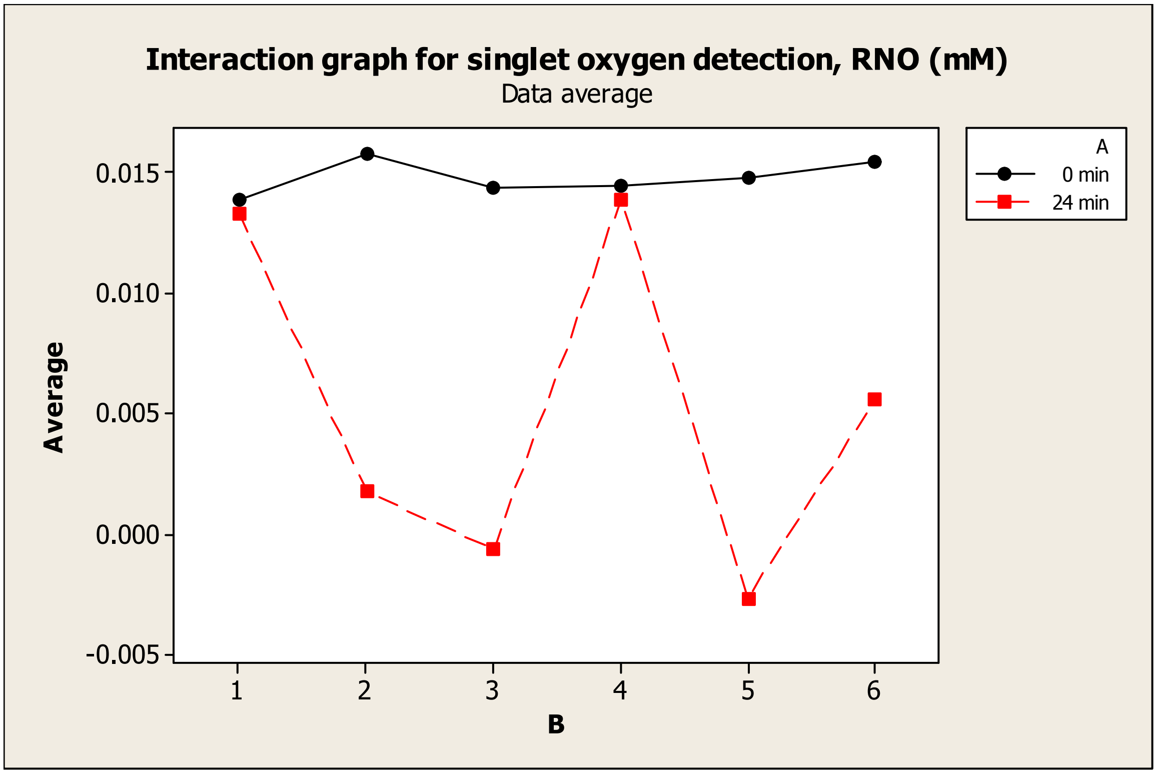

Fig. 11S Interaction graph for singlet oxygen detection test. 1=control, 2=P25, 3=P25-M, 4=G, 5=1:3-M y 6=1:3-I.

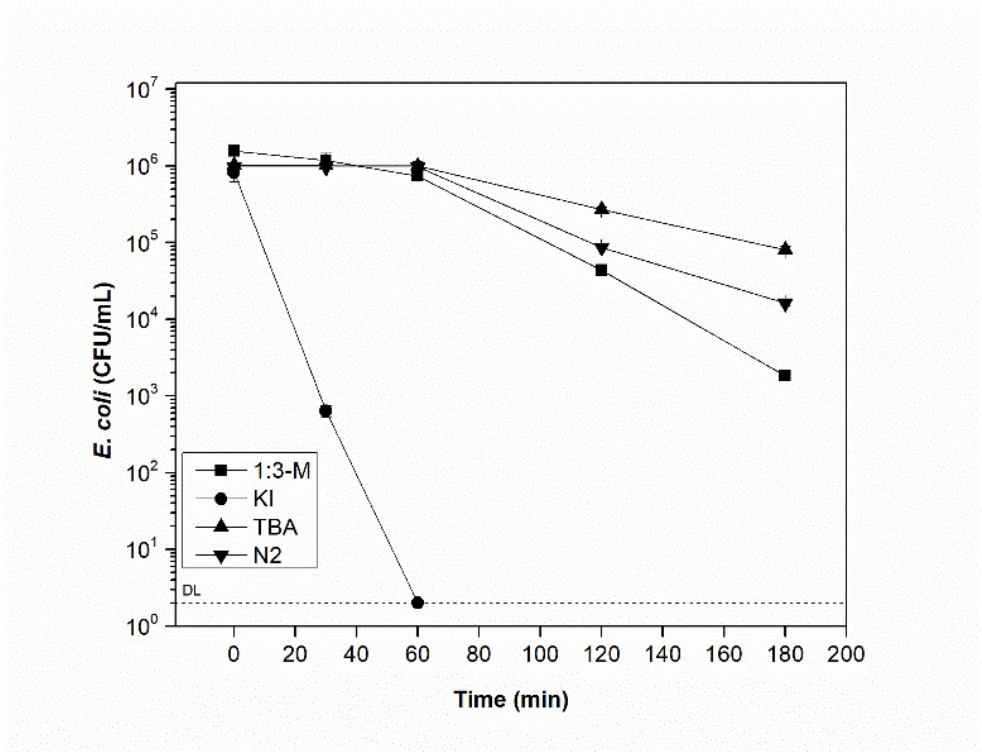

Fig. 12S. Scavengers study under visible irradiation with 1:3-M catalyst.

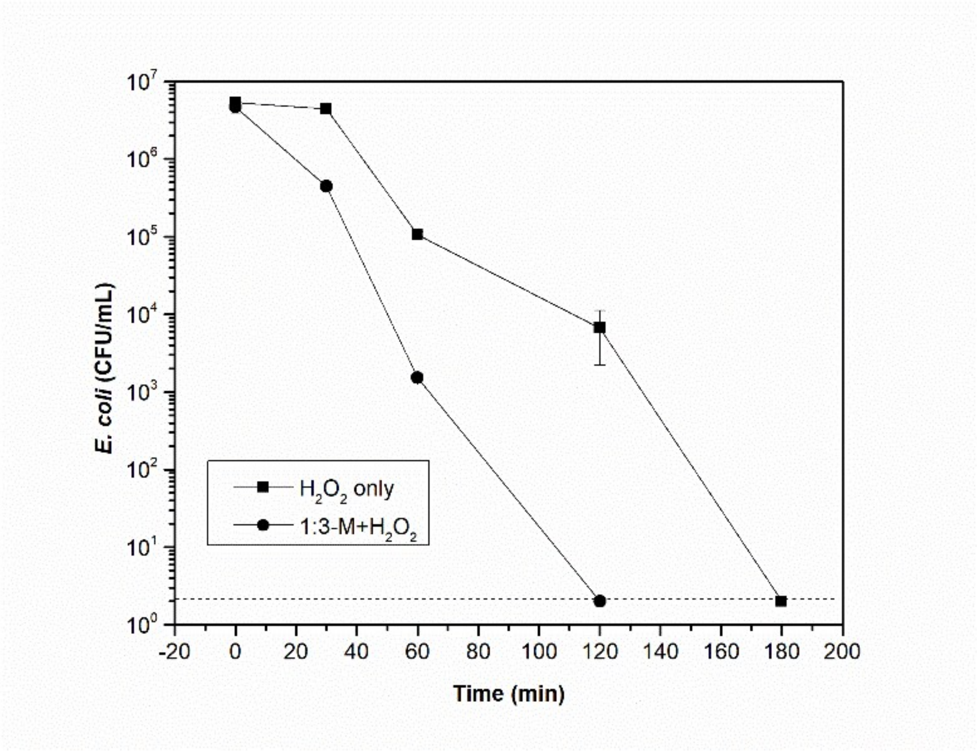

Fig. S13. 1:3-M (300 mg/L) and H_2O_2 (10 mg/L) as electron acceptor in *E. coli* disinfection under visible irradiation.
